# The Mitogenomic Characterization and Phylogenetic Analysis of the Plant Pathogen *Phyllosticta yuccae*

**DOI:** 10.3390/genes15010111

**Published:** 2024-01-17

**Authors:** Hui Xu, Ziyi Zhu, Zeyuan Tian, Cuiyuan Wei, Qi Fan, Yuanbing Wang, Shikang Shen, Gang Deng, Mingliang Ding

**Affiliations:** 1 School of Agriculture, Yunnan University, Kunming 650091, China; xhui@mail.ynu.edu.cn (H.X.);; 2 Yunnan Key Laboratory for Fungal Diversity and Green Development, Kunming Institute of Botany, Chinese Academy of Sciences, Kunming 650201, China; 3 CAS Key Laboratory for Plant Diversity and Biogeography of East Asia, Kunming Institute of Botany, Chinese Academy of Sciences, Kunming 650201, China; 4 College of Life Science and Technology, Guangxi University, Nanning 530004, China; 5 School of Ecology and Environmental Science, Yunnan University, Kunming 650091, China; 6 Food Crops Research Institute, Yunnan Academy of Agriculture Sciences, Kunming 650200, China; 7 Department of Plant Pathology, College of Plant Protection, China Agricultural University, Beijing 100083, China

**Keywords:** *Phyllosticta yuccae*, mitochondrial genome, phylogenetic analysis

## Abstract

*Phyllosticta yuccae* is an important plant pathogen causing leaf spot disease in *Yucca gigantea* Lem. It is imperative to note that the amount of information available about the mitogenome of this subject is severely limited. This must be addressed immediately, as it is crucial to our understanding and progress in this field. To better understand the mitogenomic characteristics of *P. yuccae*, we conducted its sequencing by MGISEQ. Afterwards, the mitogenome was assembled and annotated. The mitogenomic characteristics and phylogenetic placement of the *P. yuccae* strain KUMCC 6213 were analyzed. The study revealed that the mitogenome of *P. yuccae* is a circular DNA molecule, consisting of 178,540 base pairs. It contains a total of 64 genes, including 14 protein-coding genes (PCGs), 26 transfer RNA genes (tRNA), 2 ribosomal RNA genes (rRNA), and 22 open reading frame genes (ORF), accounting for 80.98% of the total size. Repetitive sequences accounted for 15.42% of the mitogenome. The analysis of codon usage indicated that the codon UUA was the most commonly utilized, whereas the amino acid Leu was the most frequently employed. A comparative analysis of mitogenomes between *P. yuccae* and *Macrophomina phaseolina* (Tassi) Goid. showed notable variations in the position and size of gene clusters, with cox1, nad4, and nad4L genes exhibiting relatively low conservation. Phylogenetic analysis based on the 14 PCGs revealed that *P. yuccae* has the closest genetic relationship with *M. phaseolina* (Botryosphaeriaceae, Botryosphaeriales). This study first reports the mitogenome of *P. yuccae* and validates its phylogenetic placement. The findings enhance the knowledge of mitogenomes in Botryosphaeriales, offering novel perspectives on the genetics and evolution of the plant pathogen *P. yuccae*. This is crucial for the accurate prevention and management of leaf spot disease in *Y. gigantea.*

## 1. Introduction

Mitochondria, which are essential organelles in most eukaryotic cells, play a major role in generating cellular energy, and serve as the primary sites for aerobic respiration [1]. They perform crucial functions in important biological processes, such as cellular senescence and apoptosis [2]. The mitochondrial genome is an effective tool for accurately identifying species across different biological taxa in molecular systematics and evolutionary biology, due to its small size and matrilineal mode of inheritance [3,4]. Currently, mitogenomes are widely used in molecular systematics and evolutionary biology [5,6,7]. However, due to the limited data on fungal mitogenomes, applications in fungi have been restricted [8]. Nevertheless, with the increasing availability of data, the use of mitogenomes in fungi is gradually expanding. These applications currently encompass not only the identification and classification of species, but also the research of disease-causing potential, studies on resistance to drugs, and the advancement of tools for diagnosing fungal infections [9,10].

The mitogenomes of eukaryotes exhibit great diversity in organizations, gene contents, and structures. Animal mitogenomes are typically small and compact in structure, with highly conserved genes, whereas plant mitogenomes are large and contain large intergenic repetitions. Fungal mitogenomes are intermediate in size, generally consist of 14 conserved core protein-coding genes (PCGs), two ribosomal RNA genes, and multiple tRNA genes. They are characterized by variable gene contents, abundant self-splicing introns, and unique mechanisms of replication and transcription [11,12]. Fungal mitogenomes vary in size, gene structure, and arrangement. Typically, their size is highly correlated with the number of repeats and introns [13,14,15]. Therefore, understanding the structural characteristics of fungal mitogenomes and the variations between homologous species can aid in the analysis of molecular evolutionary and phylogenetic patterns [16]. The study by Fonseca et al. analyzed numerous fungal mitogenomes and revealed significant variations in their size and composition, thus offering novel insights into the diversity and evolutionary trajectories of fungal mitogenomes [8].

In recent years, the development of high-throughput sequencing technology has led to a significant increase in the number of fungal mitochondrial genome databases. This has greatly facilitated research into the phylogeny and evolution of fungi [17]. To date, 1242 fungal mitochondrial genomes data have been released from NCBI Organelle database (https://www.ncbi.nlm.nih.gov/genome/browse#!/organelles/, accessed on 12 April 2023), including 38 species of Dothideomycetes. However, mitogenomes of Phyllosticaceae species have not been reported yet.

*P. yuccae* Bissett. belongs to the family Phyllosticaceae (Dothideomycetes, Botryosphaeriales), and is a significant plant pathogen. This pathogen causes leaf spot disease in *Y. gigantea*, which can harm the plant’s appearance, growth, and survival. The Canadian Department of Agriculture reported the pathogen on *Y. gigantea* leaves in 1979 [18]. In a pivotal study in 2013, Wikee et al. adopted DNA data from the ITS, LSU, ACT, TEF, and GPDH genes to redefine the genus *Phyllosticta*, and determined that *P. yuccae* belonged to the family Phyllosticaceae [19]. In this study, we first assembled and annotated the mitogenome of *P. yuccae*, which belongs to the family Phyllosticaceae of Botryosphaeriales. The analysis included an examination of its mitogenome composition, the secondary structure of tRNA genes, and codon usage. Additionally, we conducted a comparative analysis of the mitogenome of *P. yuccae* and *M. phaseolina*, and constructed a phylogenetic tree using the 14 PCGs to determine the phylogenetic position of *P. yuccae*. Our research on the mitogenome of *P. yuccae* may offer new insights into the evolutionary biology and phylogeny of fungi that are harmful to plants.

## 2. Materials and Methods

### 2.1. Sampling, Library Preparation and Sequenceing

We collected samples of diseased leaves from *Y. gigantea* plants at Qingxiu Mountain, Nanning City, Guangxi Province, China, located at 108.37° E longitude and 22.79° N latitude. We took samples from infected leaves by cutting them into small 3–5 cm segments. To prepare the samples, we treated them with 1.5% sodium hypochlorite solution for two minutes, soaking them in 70% ethanol for two minutes, rinsed them three times with sterile distilled water, and then dried them with sterile filter paper [20]. We first sterilized some leaves and cut them into small pieces using sterile scissors and forceps. These slices were then placed on potato dextrose agar (PDA) medium that contained ampicillin and streptomycin. Each Petri dish had about five to six pieces and was kept in an incubator at a steady temperature of 20 °C. After 2 to 8 days, the strains were transferred to fresh PDA and again incubated at 20 °C until pure cultures were obtained. We followed the methods described by Wang et al. to extract DNA and amplify ITS sequences [21]. Through a phylogenetic tree analysis based on the ITS data (Supplemental Figure S1), we were able to identify the strain as *P. yuccae*. The ITS sequence has been submitted to GenBank and can be accessed under the accession number OQ608827. The fungal strain KUMCC 6213, isolated from *Y. gigantea* infected leaves, is currently preserved at the Kunming Institute of Botany Culture Collection (KUMCC), Chinese Academy of Sciences, China.

We sent mycelia cultured on PDA at 20 °C for 15 days to Wuhan Huada Co. (Wuhan, China) Genomic DNA was extracted using the MGIEasy Universal DNA Library Preparation Kit (MGI Tech Co., Ltd., Shenzhen, China). This DNA was fragmented using Covaris ultrasonication. After that, DNA libraries were created through several steps including end repair and A-tail modification, sequencing aptamer ligation, purification, and PCR amplification. The concentration of the libraries was assessed using Qubit 2.0, and the insert fragments were analyzed with the Agilent 2100 Bioan-alyzer. Finally, genome sequencing was performed on the MGISEQ-2000RS platform, utilizing 2 × 150 bp paired-end read sequencing technology [22].

### 2.2. Mitogenome Assembly and Annotation

The GetOrganelle v1.7.7.0 was used for the de novo assembly of the mitogenome, resulting in a circular mitogenome [23]. MITOS (http://mitos2.bioinf.uni-leipzig.de/index.py, accessed on 20 March 2023) and MFannot (https://megasun.bch.umontreal.ca/apps/mfannot/, accessed on 20 March 2023) were used for annotation, based on the mold mitogenome genetic code [24,25]. We utilized the annotation information of *M. phaseolina* (GenBank number: NC_060329) as a reference [26]. To obtain complete annotation information, we performed multiple rounds of manual correction. The annotated mitogenome of KUMCC 6213 has been submitted to GenBank under the accession number OQ621729. To map the mitogenome, we used the online tool Chloroplot (https://chlorobox.mpimp-golm.mpg.de/OGDraw.html, accessed on 25 March 2023) [27].

### 2.3. Mitogenome Characteristics Analysis

The mitogenome of *P. yuccae* was analyzed using Geneious v9.2.0. The GC shift was assessed using the formula GC skew = (G − C)/(G + C). Codon usage analysis was analyzed with MEGA v7. [28]. The tRNA genes’ secondary structure was predicted using tRNAscan-SE v2.0. [29]. We used Mauve v2.4.0 to compare the collinearity of mitogenomes between *P. yuccae* and *M. phaseolina* [30]. In order to detect repetitive sequences in the mitochondrial genome, we used multiple tools: REPuter (https://bibiserv.cebitec.uni-bielefeld.de/reputer, accessed on 23 April 2023) for predicting dispersed repeats [31], Tandem Repeats Finder (https://tandem.bu.edu/trf/trf.html, accessed on 23 April 2023) for tandem repeats [32], and MISA (https://webblast.ipk-gatersleben.de/misa/, accessed on 24 April 2023) for simple repeats [33]. Synonymous (KS) and non-synonymous (KA) substitutions in protein-coding genes were calculated using KaKs_Calculator 2.0 [34].

### 2.4. Phylogenetic Analysis

We analyzed the genetic makeup of *P. yuccae* using 14 PCGs from 28 species. We combined the mitogenomes of 27 species obtained from the NCBI Organelle database with the *P. yuccae* mitogenome created in this study. *Trametes cingulata* Berk. (GenBank number: NC_013933) and *Lentinula edodes* Shimomura et al. (GenBank number: NC_018365) both from Basidiomycota were used as outgroups. The 14 PCGs were extracted and concatenated using PhyloSuite v1.2.3., and missing data in the analysis were filled with ‘?’ characters during the analysis to maintain consistency [35]. Sequence alignment was performed using MAFFT v.7. [36]. The phylogenetic framework was established using two approaches: maximum likelihood (ML) and Bayesian inference (BI). ModelFinder was used to select the best-fit edge-unlinked partition models for both ML and BI, based on the Corrected Akaike Information Criterion (AIC) [37]. The best-fit edge-unlinked partition models chosen for each gene segment are listed in Supplemental Table S1. For this analysis, we ran four Markov chains simultaneously for a total of 2,000,000 generations. Data was sampled after every 100 generations and discarded the initial 25% of each run, which served as the burn-in period. We assumed that stationarity had been reached when the estimated sample size (ESS) exceeded 100 and the potential scale reduction factor (PSRF) approached 1.0. Subsequently, the remaining trees were used to compute the Bayesian posterior probabilities (BPP) in a 50% majority-rule consensus tree [38]. The IQ-TREE v. 2.1.3 software was used to perform maximum likelihood analysis with a best-fit edge-unlinked partition model, and we conducted 1000 ultrafast bootstrap [39]. The phylogenetic trees obtained from our analysis were initially visualized and meticulously refined using FigTree v. 1.4.4, and further edited for clarity and visual appeal using Adobe Illustrator CS6.0.

## 3. Results

### 3.1. The Characteristics of P. yuccae

**Figure 1:**
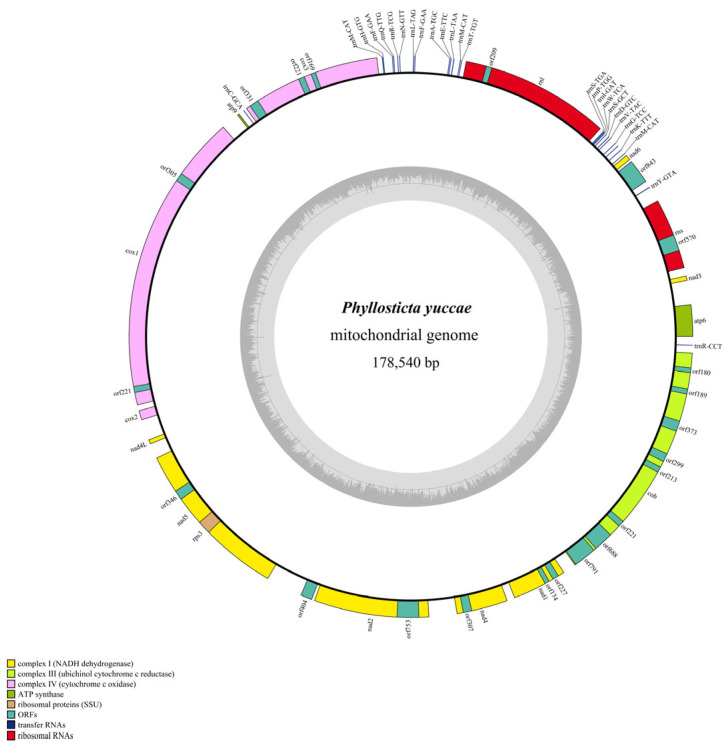
Circular map of the mitogenome of *P. yuccae*. Genes are represented by different colored blocks, as shown in the legend at the bottom left of the map.

The mitogenome of *P. yuccae* consists of a closed circular DNA molecule that measures 178,540 base pairs in length, with the coding region accounts for 80.98% of its total size (Figure 1). The G+C content of the mitogenome is 31.13%, while the A+T content is 68.87%, indicating a clear AT bias (Table 1). The mitochondrial genome contains a total of 64 genes, including 14 protein-coding genes (PCGs), 2 ribosomal RNA (rRNA) genes, 26 transfer RNA (tRNA) genes, and 22 open reading frames (ORFs). The mitogenome of *P. yuccae* is composed of repetitive sequences that make up 15.42% of its total size (Table 2, Supplemental Table S2). According to REPuter prediction, the dispersed repeat sequences had 310 repetitive units with a cumulative total length of 25,890 bp, which accounted for 14.77% of the entire mitogenome (Supplemental Table S3). There were 14 tandem repeat sequences, totaling 848 bp, which made up 0.47% of the entire mitogenome (Supplemental Table S4).

### 3.2. Protein-Coding Genes and Codon Usage

**Figure 2:**
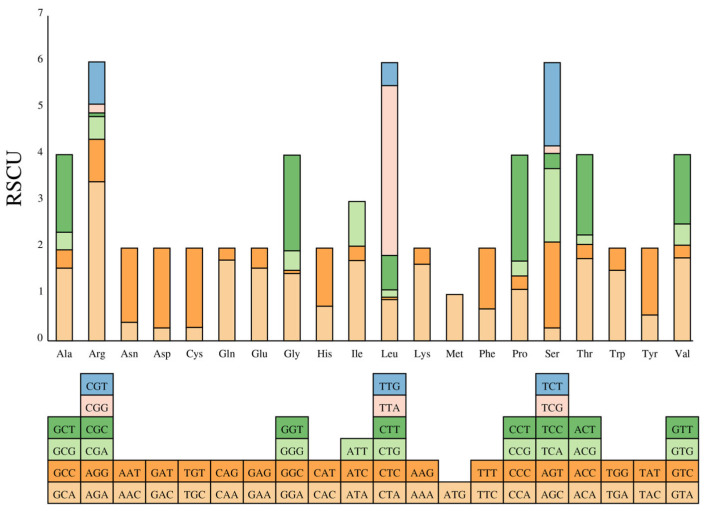
Codon usage in the mitogenome of *P. yuccae*. Codon families are indicated below the *x*-axis. RSCU is plotted on the *y*-axis. Different colors within each bar represent the different codons that encode the same amino acid.

The mitogenome of *P. yuccae* comprises 14 PCGs, which cover 9.23% of the total length, including 13 conserved core PCGs and 1 ribosomal protein S3 gene (Table 2). These 13 genes are responsible for encoding different subunits of important cellular components such as the F0 portion of the ATP synthase complex (atp6 and atp9), three cytochrome oxidase enzymes (cox1, cox2, and cox3), seven subunits of Complex I of the electron transport chain (nad1, nad2, nad3, nad4, nad4L, nad5, and nad6), and one subunit of Complex III (cob). Ribosomal protein S3 (rps3) is a component of the translation-related 40S ribosomal subunit. Atp6, nad3, and rps3 use GTG as their initiation codon, while the remaining 10 PCGs use ATG. (Table 2). The analysis of codon usage in the 14 protein-coding genes (PCGs) of *P. yuccae* showed that 5492 codons were used, and the most frequently used codon was UUA, which was used 467 times. The relative synonymous codon usage (RSCU) analysis indicated that the order of the most frequently used codons was UUA (3.66), AGA (3.43), CCU (2.28), and GGU (2.06). Furthermore, the usage frequency of amino acids in the coded proteins of the *P. yuccae* mitogenome varied greatly, with Leu being the most frequently used at 13.95% (Figure 2; Supplemental Table S5).

### 3.3. Ribosomal and Transporter RNA Genes

Ribosomal RNA is an essential component of ribosomes that plays a vital role in protein synthesis. The mitogenome of *P. yuccae* contains two rRNA genes: the small-subunit ribosomal RNA (rns) and the large-subunit ribosomal RNA (rnl). Rns is 1608 bp long and contains two introns, while rnl is 1968 bp long and contains seven introns (Table 2).

**Figure 3:**
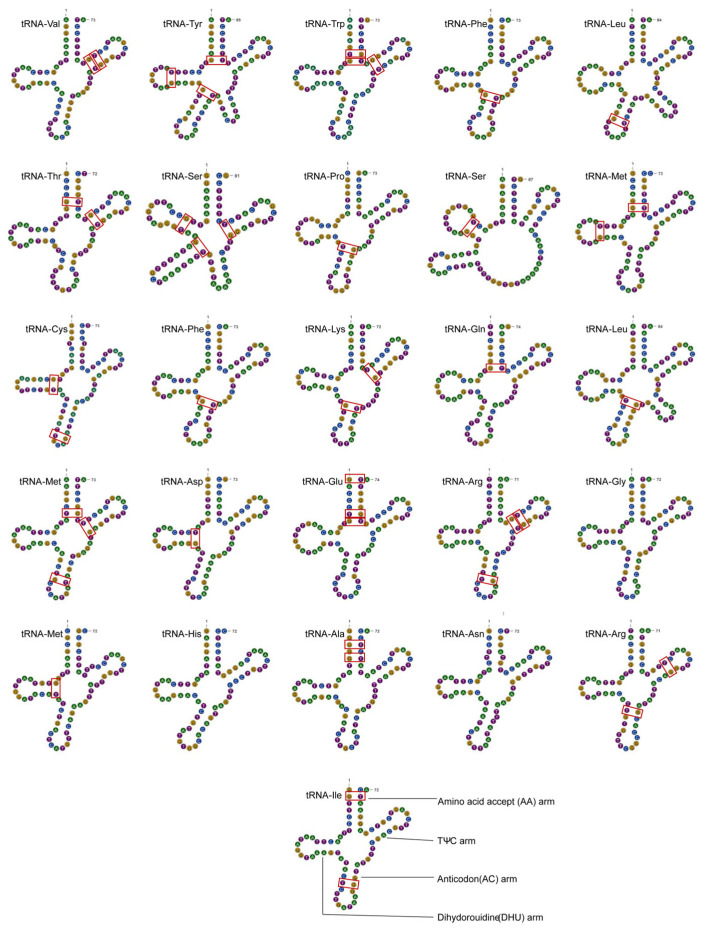
Putative secondary structures of the 26 tRNA genes identified in the mitogenome of *P. yuccae*. The red boxes represent the locations of G-U mismatches in the structure.

*P. yuccae* contains 26 tRNAs encoding amino acids. All tRNAs have a typical cloverleaf secondary structure, except for one Tyr, two Leu, and two Ser tRNAs, in which 43 G–U mismatches were identified (Figure 3). The arrangement of most transfer RNAs (tRNAs) around the rnl region in this fungal mitogenome is similar to that observed in other fungal mitogenomes, as shown in Figure 1. The tRNA genes have a combined length of 1944 base pairs, which makes up about 1.09% of the total length of the mitogenome. The length of individual tRNAs ranges from 71 to 87 base pairs (Supplemental Table S1), which was mainly due to variations in the sizes of the extra arms. Arginine (Arg), Leucine (Leu), and Serine (Ser) are transfer RNAs (tRNAs) that are encoded by tRNA genes having two different anticodons. However, Phenylalanine (Phe) is encoded by two tRNA genes that have the same anticodon, and Methionine (Met) is encoded by three tRNA genes which also have the same anticodon (Supplemental Table S2).

### 3.4. A Comparative Analysis of the Mitogenomes of P. yuccae and M. phaseolina

**Figure 4:**
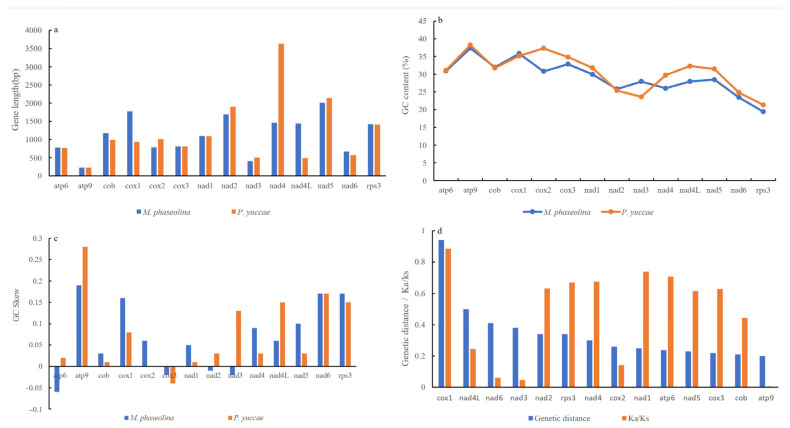
Gene analysis of 14 protein-coding genes (PCGs) in *P. yuccae* and *M. phaseolina* mitogenomes. (**a**) Length variation; (**b**) GC content; (**c**) GC skew; (**d**) genetic distance and Ka/Ks.

The mitogenomes of *P. yuccae* and *M. phaseolina*, which were found to be the most closely related species based on published data, were analyzed for collinearity using Mauve. The analysis revealed notable differences in the location and size of gene clusters between the two species (Supplemental Figure S2). The 14 conserved protein-coding genes (PCGs) showed significant differences in their size, with cox1, nad4, and nad4L having larger sizes compared to the other PCGs. In *M. phaseolina*, the lengths of cox1, nad4, and nad4L were 1776 bp, 1464 bp, and 1434 bp, respectively. In contrast, in *P. yuccae*, cox1 was 936 bp, nad4 was 3633 bp, and nad4L was 489 bp long (Figure 4a). It appears that the lengths of the cox1, nad4, and nad4L genes are not well conserved. The GC content of the 14 PCGs ranges from 31.13% in *P. yuccae* to 29.8% in *M. phaseolina*. (Table 1). In addition, there were differences in the GC content of the individual protein-coding genes (PCGs) between the two species, with atp9 having the highest GC content in both species, as shown in Figure 4b. The analysis of the *P. yuccae* mitogenome revealed that most of the PCGs had a positive GC skew, except for cox3, which had a negative GC skew. Conversely, the atp6, cox3, nad2, and nad3 of *M. phaseolina* displayed a negative GC skew, as illustrated in Figure 4c. It was discovered that the substitution rates of different PCGs vary between *P. yuccae* and *M. phaseolina*. The genetic distance analysis revealed that the largest genetic distance can be found in cox1, followed by nad4L (as shown in Figure 4d). The atp9 gene exhibited the smallest genetic distance among all the PCGs, which is an interesting finding. Moreover, the Ka/Ks values of the 14 PCGs were all found to be below one, indicating strong negative selection (as depicted in Figure 4d).

### 3.5. Phylogenetic Relationships

**Figure 5:**
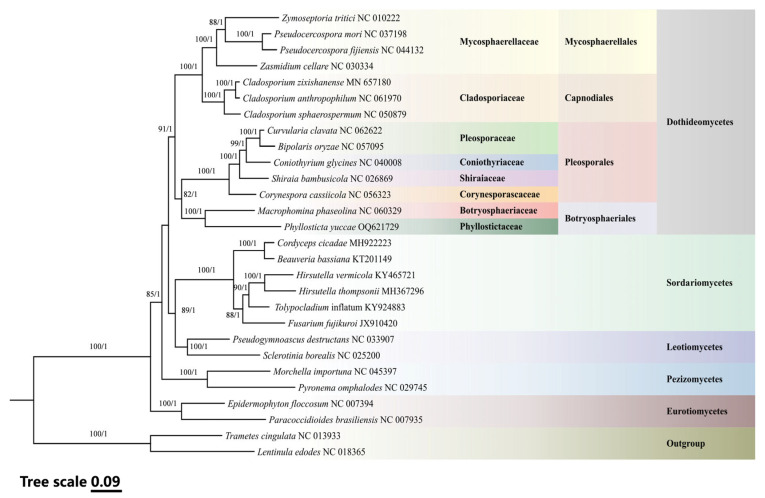
Integrated phylogenetic analysis of 28 fungal species based on Maximum Likelihood and Bayesian Inference from 14 protein-coding genes. The nodes are annotated with bootstrap support values from Maximum Likelihood analysis and posterior probabilities from Bayesian Inference analysis. Species and NCBI accession numbers for mitogenomes used in the phylogenetic analysis are provided in Supplemental Table S7.

In our study, we analyzed the mitogenomes of Ascomycota and conducted a phylogenetic analysis of *P. yuccae*, due to the limited information available on the mitogenomes of Phyllostictaceae family. We utilized Maximum Likelihood (ML) and Bayesian Inference (BI) methods. The results of the analysis showed that the machine learning (ML) and Bayesian inference (BI) trees were topologically consistent, with 14 species of Dothideomycetes forming a large clade. Within this clade, *P. yuccae* was identified as a sister taxon to *M. phaseolina*, suggesting a close evolutionary connection between them. The study found that the other 12 Ascomycota species could be grouped into four different clades, which represented four distinct classes: Leotiomycetes, Eurotiomycetes, Sordariomycetes, and Pezizomycetes. These findings not only show where *P. yuccae* fits within Ascomycota, but also highlight the diversity and evolutionary relationships within Dothideomycetes. The high bootstrap values in our ML tree and strong posterior probabilities in our BI tree confirm the reliability of the phylogenetic tree, providing compelling support for our conclusions. The results of our study are represented in a clear manner through Figure 5. Additionally, we have provided detailed mitochondrial genomic data in Supplemental Table S7 for further analysis and validation. These data can be utilized by future studies to explore the evolutionary relationships of other unclassified groups within Ascomycota. By doing so, we can gain a deeper understanding of the genetic diversity and evolutionary history of this clade.

## 4. Discussion

In this study, we present the complete mitogenome of the plant pathogen *P. yuccae* in the Phyllosticaceae. The mitogenome of *P. yuccae* is the largest in Botryosphaeriales, at 178,540 bp (Figure 1). The coding region of this organism’s mitogenome makes up 80.98% of the total and contains a total of 64 genes. Out of the 14 protein-coding genes (PCGs), atp6, nad3, and rps3 use GTG as the starting codon, whereas the other 10 PCGs start with the conventional ATG codon (Table 2). Although ATG is a typical starting codon in eukaryotes, alternative codons can also initiate protein synthesis [40,41,42]. Using a homologous codon for translation initiation may enhance coding potential or have regulatory functions [43]. Fungal mitogenomes have varying sizes; for example, *Rozella allomycis* has a mitogenome size of only 12 kb (GenBank number: NC_021611) [44], whereas the largest fungal mitogenome on record is that of *Golovinomyces cichoracearum*, which has a length of 332.125 kb (GenBank number: NC_056148) [45]. Previous studies suggest that repetitive sequences, intergenic regions, the number of ORFs, and horizontal gene transfer primarily influence fungal mitogenome size variation [46]. The mitogenome of *P. yuccae* is 178,540 bp, while that of its relative *M. phaseolina* is 101,198 bp. Repeats and intergenic regions in *P. yuccae* are approximately twice as long as those in *M. phaseolina* (Table 1). Thus, the primary factors contributing to the length variation of mitogenomes in Botryosphaeriales may be the lengths of repeats and intergenic regions. A comparison of mitochondrial gene arrangements can provide valuable insights into species evolutionary relationships [47,48]. In this study, significant rearrangements were observed in the mitogenomes of two Botryosphaeriales species, involving core PCGs, ORFs, tRNA genes, and rRNA genes. Among the eight gene clusters detected, seven showed changes in their locations (Supplemental Figure S2). Previous studies have shown that the accumulation of repetitive sequences in fungal mitogenomes is closely associated with mitochondrial gene rearrangements and gene loss [17,49]. In terms of repetitive sequences, *P. yuccae*’s mitogenome consists of 15.4%, while *M. phaseolina*’s mitogenome consists of 12.27% (Table 1). This suggests that during fungal evolution, while most mitochondrial genes have been transferred to the nuclear genome, fungi generally retain 14 key genes related to metabolic energy in their mitochondria. As a result, both species have undergone significant gene rearrangements [50]. During our comparison of the mitogenome of *P. yuccae* and *M. phaseolina*, we discovered that 13 PCGs in *P. yuccae* had a positive GC skew, except for cox3, which had a negative skew. On the other hand, in *M. phaseolina*, the GC skew of atp6, cox3, nad2, and nad3 was negative (Figure 4c). Some studies suggest that base bias may result from asymmetric base mutations and different selection pressures encountered during replication and transcription [51,52]. Additionally, the genetic distances of the 14 protein-coding genes (PCGs) are distinct, suggesting that different PCGs in Botryosphaeriales have varying evolutionary rates (Figure 4d). Botryosphaeriales exhibit high conservation in atp9, with all 14 PCGs evolving under purifying selection (Ka/Ks < 1) (Figure 4d).

Our phylogenetic analysis, based on 14 PCGs from mitogenomes of 28 species, yielded topological structures consistent with previous studies [19,26]. At the base of the phylogenetic tree is Basidiomycota, while a large clade includes 14 species of Dothideomycetes. Based on the analysis, it has been identified that *P. yuccae* and *M. phaseolina* are closely related to each other, with a high level of genetic similarity. The node that connects them has received a bootstrap support value of 100 from ML and a posterior probability of 1.0 from BI, providing strong evidence for their genetic relationship. Additionally, the remaining 12 Ascomycota species are categorized into four distinct clades: Leotiomycetes, Eurotiomycetes, Sordariomycetes, and Pezizomycetes (Figure 5; Supplemental Table S7). The analysis of the genetic similarity between *P. yuccae* and *M. phaseolina* shows that they have relatively short homologous regions and poor collinearity. This is likely due to the fact that they belong to two different families and are still genetically distant. These findings indicate that differences in homologous regions may be the primary cause of variations among fungal species. Currently, there is limited mitogenomic data available for the Botryosphaeriales order, which hinders our comprehensive understanding of species diversity within this order. Future research should expand the mitogenomic database for Botryosphaeriales by incorporating more species to enrich our phylogenetic framework and explore genetic variations. Such efforts will enhance our understanding of fungal differentiation and adaptation throughout their evolutionary history.

## 5. Conclusions

For the first time, this study marks the assembly and annotation of the mitogenome of *P. yuccae*. With a length of 178,450 bp, including 39 introns, 398 repeats, and 22 ORFs, it is the largest reported mitogenome among Botryosphaeriales thus far. Significant differences in gene size, arrangement, and collinearity between *P. yuccae* and *M. phaseolina* suggest that interspecies variations may be related to differences in homologous regions.

In addition, phylogenetic analysis indicates that *P. yuccae* clusters with *M. phaseolina*. This study provides new evidence for the phylogenetic placement of *P. yuccae*. The comparative mitogenome analysis of *P. yuccae* and *M. phaseolina* has also enriched the understanding of mitochondrial evolution in the order Botryosphaeriales.

## Figures and Tables

**Table 1:** Comparison between *P. yuccae* and *M. phaseolina* mitogenomes.

Species		*M. phaseolina*	*P. yuccae*
Accession No.		NC_060329	OQ621729
Genome size (bp)		101,198	178,540
GC content (%)		29.8%	31.13%
PCGs	Number	14	14
	Length (bp)	15,741	16,476
	Ratio (%)	15.55%	9.23%
tRNA	Number	26	26
	Length (bp)	1940	1944
	Ratio (%)	1.92%	1.09%
rRNA	Number	2	2
	Length (bp)	4330	3576
	Ratio (%)	4.28%	2.00%
ORF	Number	38	22
	Length (bp)	37,683	24,168
	Ratio (%)	37.24%	13.54%
Intron	Number	21	39
	Length (bp)	33,788	120,240
	Ratio (%)	33.39%	64.35%
SSR	Number	34	74
	Length (bp)	427	789
	Ratio (%)	0.42%	0.44%
Tandem	Number	16	14
	Length (bp)	778	848
	Ratio (%)	0.77%	0.47%
Interspersed	Number	157	310
	Length (bp)	11,210	25,890
	Ratio (%)	11.08%	14.75%
Intergenic regions	Length (bp)	25,863	33,964
	Ratio (%)	25.56	19.02

**Table 2:** PCGs and rRNA genes composition of *P. yuccae* mitogenome.

Gene Name	Type	Position		Length	Codons		Intron Number	Introns Length	Strand
		From	To		Start	Stop			
atp6	CDS	1	3268	765	GTG	TAG	1	2503	+
nad3	CDS	5707	6210	504	GTG	TAA	-	-	+
rns	rRNA	7032	14,366	1608	-	-	2	5727	+
nad6	CDS	19,372	19,947	576	ATG	TAA	-	-	+
rnl	rRNA	23,680	39,033	1968	-	-	7	13,386	+
cox3	CDS	48,055	62,352	810	ATG	TAG	7	13,488	+
atp9	CDS	63,244	63,468	225	ATG	TAA	-	-	+
cox1	CDS	65,320	96,273	936	ATG	TAG	5	30,018	+
cox2	CDS	96,830	97,837	1008	ATG	TAG	-	-	+
nad4L	CDS	99,943	100,431	489	ATG	TAA	-	-	+
nad5	CDS	101,949	118,788	2139	ATG	TAA	6	14,701	+
rps3	CDS	109,790	111,196	1407	GTG	TAA	-	-	+
nad2	CDS	123,965	135,750	1896	ATG	TAG	4	9890	+
nad4	CDS	138,561	143,823	3633	ATG	TAG	1	1630	+
nad1	CDS	144,594	150,225	1095	ATG	TAA	2	4537	+
cob	CDS	151,541	176,893	993	ATG	TAA	4	24,360	+

## Data Availability

The genome sequence data that support the findings of this study are openly available in GenBank of NCBI under the accession number OQ621729. The associated BioProject, SRA, and Bio-Sample numbers are PRJNA950187, SRR24012831, and SAMN33977573, respectively.

## Supplementary Materials

The following supporting information can be downloaded at: https://www.mdpi.com/article/10.3390/genes15010111/s1, Figure S1: Phylogenetic tree of 23 species within Phyllosticta based on Maximum likelihood analysis of ITS gene; Figure S2: Mitogenome comparative collinearity analysis between *P. yuccae* and *M. phaseolina*; Table S1: The best-fit edge-unlinked partition model; Table S2: tRNA feature and organization in the *P. yuccae* mitogenome; Table S3: Distribution of repeat loci in the mitogenome of *P. yuccae* and *M. phaseolina* as revealed by REPuter; Table S4: Tandem repeats detected in the mitogenomes of *P. yuccae* and *M. phaseolina*; Table S5: Codon usage analysis between *P. yuccae* and *M. phaseolina*; Table S6: Microsatellite DNA in *P. yuccae* and *M. phaseolina*; Table S7: Mitogenome information used for phylogenetic analyses in this study.
