# Supplementary material for: The Mitogenomic Characterization and Phylogenetic Analysis of the Plant Pathogen Phyllosticta yuccae
Source: Genes (Basel). 2024 Jan 17;15(1):111. doi: 10.3390/genes15010111 (PMC10815617; doi:10.3390/genes15010111)
Supplement: Supplementary file 1 [file genes-15-00111-s001.zip › genes-2785523 - Supplementary Figures.pdf]

genes MDPI

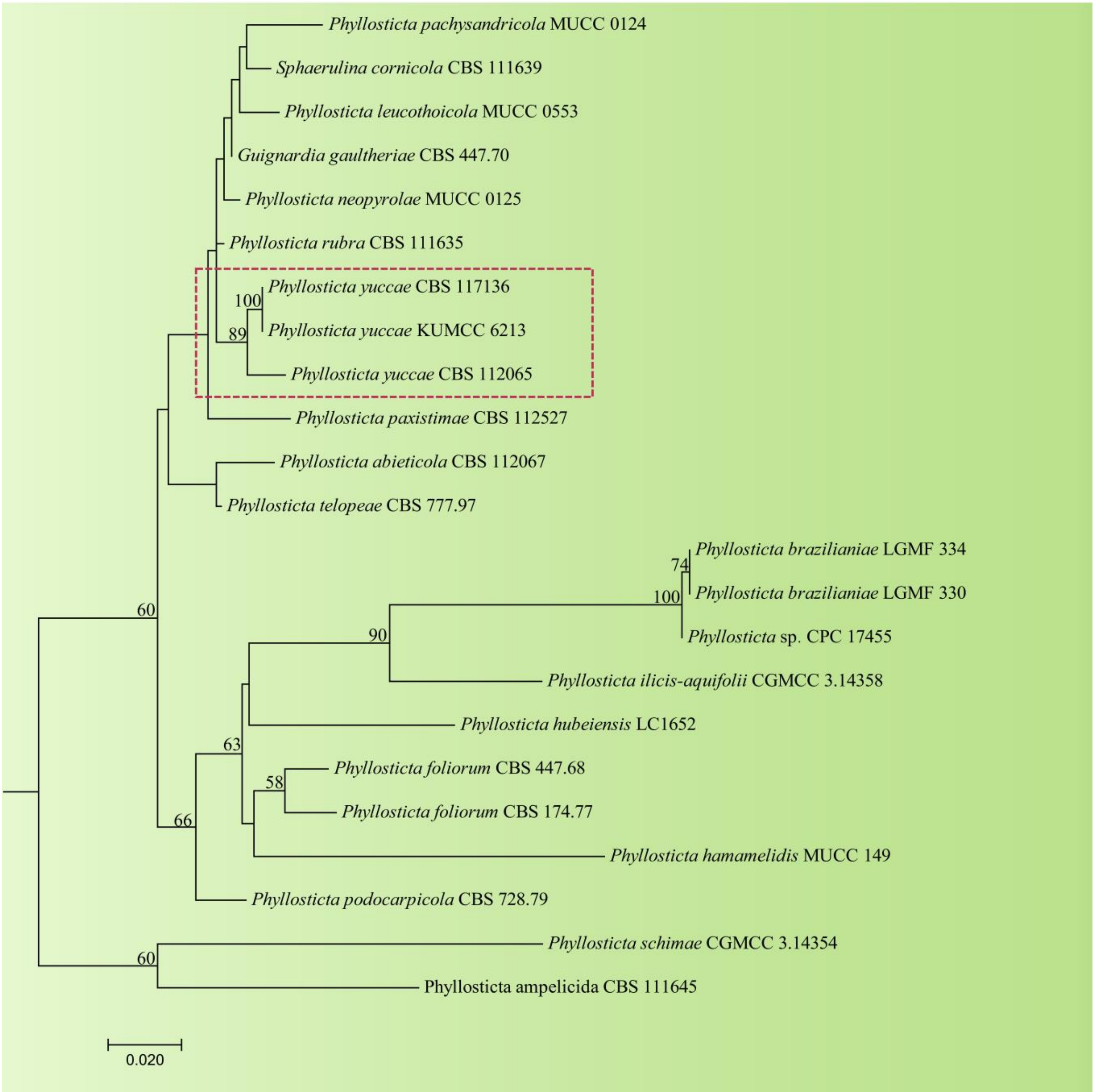

Figure S1. Phylogenetic tree of 23 species within Phyllosticta based on Maximum likelihood analysis of ITS gene.

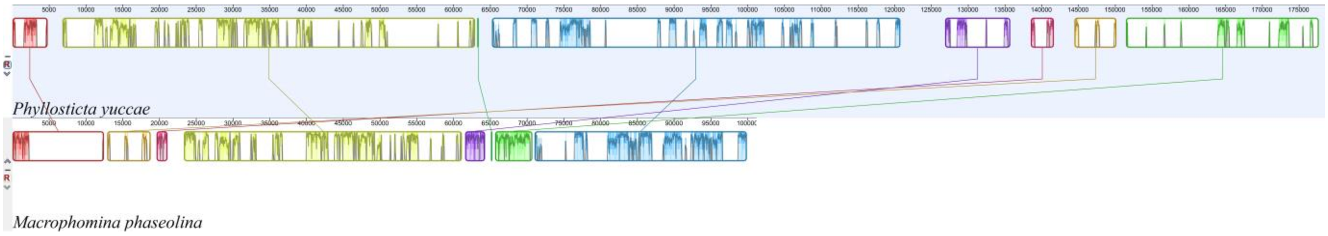

Figure S2. Mitogenome comparative collinearity analysis between *P. yuccae* and *M. phaseolina*.
